# 1,3,4-Thiadiazole Derivatives. Part 9^1^. Synthesis and Biological Activity of Metal Complexes of 5-(2-Aminoethyl)-2-Amino-1,3,4-Thiadiazole

**DOI:** 10.1155/MBD.1996.227

**Published:** 1996

**Authors:** Mihai Barboiu, Marilena Cimpoesu, Cornelia Guran, Claudiu T. Supuran

**Affiliations:** 1 Research Center for Macromolecular Materials and Membranes Spl. Independentei 206 Bucharest 79611 Romania; 2 University of Bucharest Department of Inorganic Chemistry Str. Dumbrava Rosie 23 Bucharest Romania; 3 Polytechnic University of Bucharest Department of Inorganic Chemistry, Polizu 1 Bucharest 79611 Romania; 4 Università degli Studi Laboratorio di Chimica Inorganica e Bioinorganica Via Gino Capponi 7 Florence I-50121 Italy

## Abstract

Metal complexes of the title ligand (L) containing Co(II), Ni(II) and Cu(II) were prepared and characterized by elemental analysis, IR, electronic spectroscopy and conductimetry. The new derivatives, possessing the following formulae, CuL_2_(OH)_2_, NiL_2_Cl_2_, and [Co_2_LCl_4_]_n_ showed *in vitro* antifungal activity against *Aspergillus* and *Candida spp.*


### 1,3,4-THIADIAZOLE DERIVATIVES. PART 9[1]. SYNTHESIS AND BIOLOGICAL ACTIVITY OF METAL COMPLEXES OF 5-(2-AMINOETHYL)-2-AMINO-1,3,4-THIADIAZOLE

Mihai Barboiu[1], Marilena Cimpoesu[2], Cornelia Guran[3], and Claudiu T. Supuran[4*]

[1] Research Center for Macromolecular Materials and Membranes, Spl. Independentei 206, 79611 Bucharest, Roumania
[2] University of Bucharest, Department of Inorganic Chemistry, Str. Dumbrava Rosie 23, Bucharest
[3] Polytechnic University of Bucharest Department of Inorganic Chemistry, Polizu 1, 79611 Bucharest, Roumania
[4] Università degli Studi, Laboratorio di Chimica Inorganica e Bioinorganica, Via Gino Capponi 7, I-50121, Florence, Italy

**Abstract**: Metal complexes of the title ligand (L) containing Co(II), Ni(II) and Cu(II) were prepared and characterized by elemental analysis, IR, electronic spectroscopy and conductimetry. The new derivatives, possessing the following formulae, $CuL_2(OH)_2$, $NiL_2Cl_2$, and $[Co_2LCl_4]_n$ showed *in vitro* antifungal activity against *Aspergillus* and *Candida spp*.

### Intoduction

1,3,4-Thiadiazole derivatives possess interesting biological activity probably conferred to them by the strong aromaticity of this ring system,[2] which leads to great *in vivo* stability and generally, a lack of toxicity for higher vertebrates, including humans. When diverse functional groups that interact with biological receptors are attached to this ring, compounds possessing oustanding properties are obtained. Except for some antibacterial sulfonamides (albucid and globucid), no longer used clinically, but which possessed historical importance,[2] the most interesting examples are constituted by 5-amino-1,3,4-thiadiazole-derivatives such as the thiol **1**, a compound used as radioprotective agent,[3] as well as an investigational antitumor[4] and gastroprotective[5] drug; acetazolamide **2**, which was the first non-mercurial diuretic drug,[6-9] used clinically thereafter as antiglaucoma,[10] antiepileptic[11] or antiulcer drug,[12] together with a large series of its congeners derived from 5-amino-1,3,4-thiadiazole-2-sulfonamide **3**.[9]

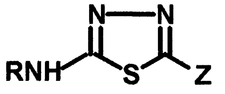

**1**: R=H, Z=SH
**2**: R=Ac, Z=$SO_2NH_2$
**3**: R=H, Z=$SO_2NH_2$

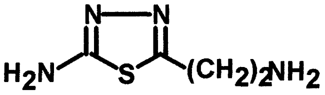

**4**

These compounds, have also been investigated as complexing agents,[13-16] in order to obtain substances with a diversified biological activity, conferred to them among others by the presence of the metal ions, except for that of the thiadiazole ligand. Thus, some metal complexes of ligands of type **1-3** have been recently reported as *in vitro* inhibitors of the zinc enzyme carbonic anhydrase (CA, EC 4.2.1.1),[1,14,16] whereas *in vivo* studies showed good antiepileptic action for some Cu(II) and Zn(II) complexes of the sulfonamide type ligands.[15d] Finally, some 2,5-disubstituted-1,3,4-thiadiazoles as well as their Cu(II) complexes were reported to act as fungitoxic agents.[16h]

Recently, another group of biologically active 1,3,4-thiadiazoles was reported by us.[17] 5-(2-Aminoethyl)-2-amino-1,3,4-thiadiazole **4** and some of its positively charged pyridinium derivatives showed very good activatory effects on carbonic anhydrase,[17] making such compounds attractive candidates for the development of pharmacological agents for the treatment of CA defficiency syndrome, a rare but dramatic genetic disease which is caused by the lack of isozyme CA II in the human organism.[18,19]

As **4** possesses an interesting donor atoms system besides its prominent biological activity,[17,19] a study for preparation and biological assay of some of its metal complexes was initiated in our laboratory. Fungitoxicity has not been investigated previously for amines of type **4**, and since many imidazoles and triazoles such as ketoconazole, fluconazole, clortrimazole, etc possess outstanding fungistatic properties, being widely used clinically,[20] it appeared of interest to test thiadiazole **4** and some of its metal complexes for this type of biological activity. In this paper we report the synthesis of Co(II), Ni(II) and Cu(II) complexes of ligand **4**, and their activity as fungistatic agents against *Aspergillus* and *Candida spp.*

### Materials and Methods

Infrared spectra were recorded in CsBr pellets with a Nicolet 2DXFT-IR apparatus. Electronic spectra were recorded by the diffuse reflectance technique using MgO as a reference material. Conductometry was done in DMF solution with a Radelkis 1200K/1 apparatus at 25°C. Elmental analyses were done by combustion (C,H,N,S) with an automated Carlo Erba analyser, or by atomic absorption spectroscopy for the metal ions. Ligand **4** was prepared as described in the literature.[17] Metal salts and solvents were analytical grade (from Merck or Aldrich) and were used without further purification.

*Synthesis of complexes* **5-7**

All complexes reported in this paper have been synthesised according to the following general procedure: methanolic solutions containing the metal chloride ($CuCl_2$ $2H_2O$, $CoCl_2$ $6H_2O$, $NiCl_2$ $6H_2O$) and the heterocyclic diamine, **4**, (L.2HCl),[17] were mixed with stirring in molar ratios of 1:1 and 1:2, respectively. The dihydrochloride of amine **4** was used in synthesis, due to its good methanol solubility.[17] The resulting mixture was treated with a stoichiometric amount of triethylamine (in order to neutralize the HCl of the dihydrochloride) and the obtained mixture was heated for 60 min. on a water bath. The complexes precipitated from these solutions, were filtered, washed with diethylether and dried in vacuum. The following coordination compounds were synthesised and characterised: $CuL_2(OH)_2$ (green blue) **5**, $NiL_2Cl_2$ (blue) **6**, and $Co_2LCl_4$ (red brown) **7**. In the above formulas L stands for the corresponding diamino ligand. It is to note that, even when working in molar ratio $MCl_x$: L of 1:1 or 1:2, only the above mentioned compounds were obtained.

***Copper (II) complex*** **5**: non-electrolyte, IR (CsBr), $cm^{-1}$, 470, 617, 763, 927, 1069, 1127, 1348, 1456, 1482, 1512, 1600, 2361, 2952. UV-VIS (MgO), $cm^{-1}x10^{-3}$ :7.0, 11.0, 15.7, 23.1, 26.3. Analysis:found, %Cu, 16.35; %C, 25.17; %H, 4.46, %N, 29.24, %S, 16.48; $CuC_8H_{18}N_8S_2O_2$; requires %Cu, 16.58; %C, 24.87; %H, 4.66, %N, 29.02, %S, 16.58.

***Nickel (II) complex*** **6**: non-electrolyte, IR (CsBr), $cm^{-1}$, 440, 624, 862, 941, 1127, 1162, 1283, 1351, 1422, 1510, 1605, 2368, 2952, UV-VIS (MgO), $cm^{-1}x10^{-3}$, 10.0, 17.5, 20.1, 25.3. Analysis:found, %Ni, 14.01; %C, 23.17; %H, 3.66, %N, 26.24, %S, 15.48; %Cl, 16.75 , $NiC_8H_{16}N_8S_2Cl_2$ ; requires %Ni, 14.11; %C, 22.96; %H, 3.82, %N, 26.79, %S, 15.31; %Cl, 16.98.

***Cobalt (II) complex*** 7: electrolyte, IR (CsBr), $cm^{-1}$, 316, 450, 526, 580, 859, 971, 1127, 1157, 1340, 1419, 1532, 1605, 2361, 2952, UV-VIS (MgO), $cm^{-1}x10^{-3}$, 9.1, 11.2, 14.3, 18.8, 21.5, 26.3. Analysis:found, %Co, 27.89; %C, 12.17; %H, 1.66, %N, 14.14, %S, 7.48; %Cl, 34.88 , $Co_2C_4H_8N_4SCl_4$; requires %Co, 29.20; %C, 11.88; %H, 1.98, %N, 13.86, %S, 7.92; %Cl, 35.15.

*Assay of fungistatic activity of compounds* **4-7**

The fungistatic activities of derivatives **4-7** were determined by a modification of the growth method[21] recently reported by us,[22] utilizing two *Aspergillus* and one *Candida spp*. The fungi were cultivated in agar plates at 25°C, in the absence and in the presence of compounds **4-7**, in concentrations ranges of $10^{-3}$ - $10^{-7}$ M (solutions in DMSO). No significant fungistatic effects were observed at concentrations of $10^{-6}$ and $10^{-7}$ M of the new compounds, except for the Cu(II) derivative **5** (the most active in the series), which showed interesting inhibitory activity in micromolar concentrations too. Percentual inhibition of growth was calculated with the following formula:[21,22]

$$\% \text{ inhibition} = 100 \text{ x } (Dc\text{-}Di)/Dc$$

where Dc represents the average diameter of the fungi colony in the control plate after 48 hours, whereas Di the same parameter in the presence of tested compound.

**Results and Discussion**

Due to the complicated donor system of the ligand **4**, at least four different more common complexation modes are probable, depicted as **I-IV** in the formulas bellow. The most favourable one for mononuclear complexes would be **II** or **IV**, due to the stability of six membered cycles formed in these cases, while **I** and **III** should lead to less stable four membered rings. From IR data discussed shortly, we ruled out coordination of types **III** or **IV**, as among others, no M-S bands were observed in the IR spectra. From the stereochemical point of view it is also hardly possible a coordination of type **IV**. Thus, as for histamine and histidine complexes, the most probable coordination mode is probably **II**, involving the aminoethyl $NH_2$ group and the endocyclic nitrogen N-4 of the heterocyclic ring.[23]

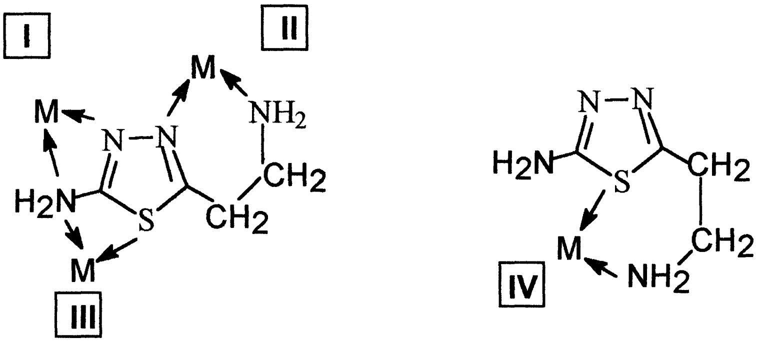

In the IR spectra of complexes **5-7** the large stretching vibrations of the ammonium bands from the IR spectrum of **4** (as dihydrochloride salt),[17] around 3000 $cm^{-1}$, are absent, due to the involvement of the free $NH_2$ groups in coordinating the metal ions. Characteristic vibrations of the ligand ($\delta_{NH2}$ at 1630 $cm^{-1}$, $\nu_{C=N}$ at 1564 $cm^{-1}$ in the spectrum of **4**) are also present in the spectra of complexes **5-7**, but shifted towards lower wavenumbers as compared to the spectrum of **4**. Thus the thiadiazolic band appears at 1510 - 1532 $cm^{-1}$ ($\nu_{C=N}$) in the spectrum of these complexes. The shift with 25-35 $cm^{-1}$ towards lower wavenumbers of the $NH_2$ and C=N vibrations in the IR spectra of the complexes is indicative that the coordination of type **II** or **I** proposed above might describe the behaviour of **4** in its metal complexes. The coordination by two types of nitrogen atoms is also supported by the existence of large bands in the range 200-600 $cm^{-1}$ in the spectra of complexes **5-7**, presumably due to M-N vibrations. A band centred at 470 $cm^{-1}$ was observed for the Cu(II) complex **5**, at 440 $cm^{-1}$ for the Ni(II) complex **6** and several peaks for the Co(II) complex **8**, at 316, 450 and 526 $cm^{-1}$.[24]

Supplementary stereochemical information for the new complexes **5-7** was obtained from the absorption electronic spectra of the three compounds.

In the electronic spectrum of the Cu(II) derivative **5**, two large bands were observed: i) the first one is located in the range 5-18 x$10^3$ $cm^{-1}$ and offers information about d-d- transitions. By deconvolution using the Gauss method,[25] the following transition bands were identified, at 7 x$10^3$, 11 x$10^3$, and 15.7 x$10^3$ $cm^{-1}$, respectively. The lowest energy, according to the $d_{x^2-y^2} \Rightarrow d_{z^2}$, transitions, is characteristic for the copper (II)

ion in a (pseudo)-octahedral configuration.[25] The band appeared at a slightly higher energy due to the involvement of the 4s orbital in the coordination sphere. ii) The second transition observed in the range 18-25 $x10^3$ $cm^{-1}$ can be attributed to charge transfer bands.[25]

The diffuse reflectance spectrum of the nickel complex **6** is characteristic for a (pseudo)-octahedral arrangement with the following transition bands: $\nu_1$: 10 $x10^3$ $cm^{-1}$ ($^3T_{2g} \Leftarrow {}^3A_{2g}$), $\nu_2$: 17.5 $x10^3$ $cm^{-1}$ ($^3T_{1g}$ (F) $\Leftarrow {}^3A_{2g}$), $\nu_3$: at higher energies (charge transfer energy). After deconvolution the first band ($\nu_1$) was splitted in two components and the second band ($\nu_2$) in three components, this supplementary division being probably due to the low simetry of the ligand field and to the non-equivalence of the donor atoms.[25,26]

The electronic spectrum of the Co(II) complex **7** possibly contains two overlapped bands of two chromophores in different stereochemistries. The presence of an intense band at 15 $x10^3$ $cm^{-1}$ confirms the tetrahedral sterochemistry, while other bands at 11.2 $x10^3$, 16.3 $x10^3$, and 21.5 $x10^3$ $cm^{-1}$, respectively, are characteristic for a (pseudo)-octahedral configuration of the Co(II) ion.[27] It is difficult to formulate this compound as *octahedral cation: tetrahedral anion* complex, (due to its brown-red color characteristic of polynuclear complexes of Co(II))[25] as the presence of the tetrahedral anion $[CoCl_4]^{2-}$ would imprint an intense blue colour to it.[25,27] Thus, the polynuclear structure shown below is proposed, whereas the other two mononuclear complexes were formulated as containing octahedral Cu(II) and Ni(II) ions.

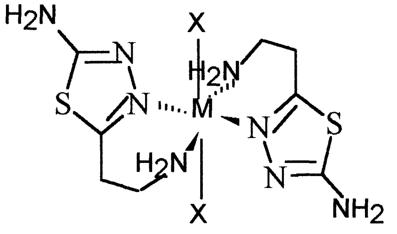

**5**: M=Cu(II), X=OH;

**6**: M= Ni(II), X=Cl

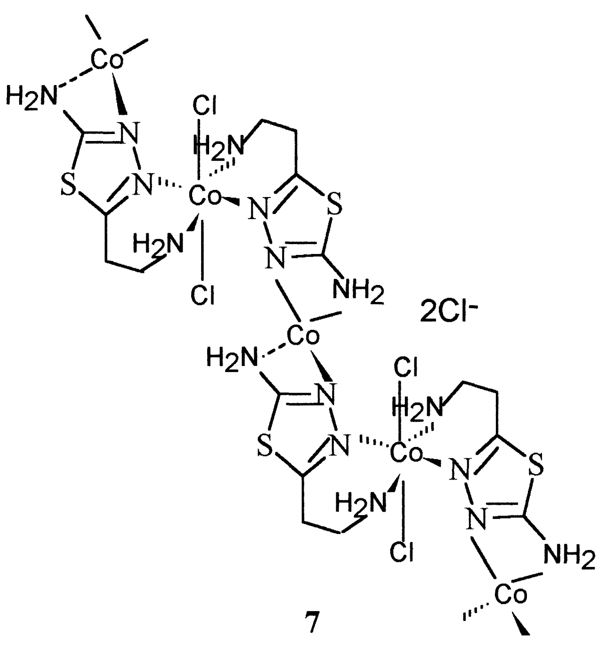

**7**

The fungistatic activities of derivatives **4-7** against two *Aspergillus* species and *Candida albicans* are presented in Table I. Clotrimazole **8**, a topically effective fungistatic agent with clinical applications was used as a standard in these assays.[20,28]

As seen from the data of Table I, the thiadiazole **4** is much less effective as fungitoxic agent as compared to clotrimazole, an imidazole derivative with a potent such activity, against all three fungi species investigated. The metal complexes **5-7** already show an increased fungitoxic activity (at the concentration of 10 μM, for which the data of Table I stand). At 0.1 - 1 μM concentrations no such activity was observed for the complexes, except for the Cu(II) derivative **5** (data not shown). At 10 μM, significant inhibitions of growth were detected for **5**, which is almost as effective as clotrimazole against *A. niger*, slightly more effective against *A. flavum*, and much less effective against *Candida*. The other two complexes showed a modest activity, which is anyhow greater against the *Aspergillus spp.* as compared to *Candida*. The metal ion inducing best fungitoxic activity was Cu(II), followed by Co(II) and Ni(II), which possessed comparative efficiency.

As for the mechanism of action of these derivatives, probably it is similar to that of the imidazole- and triazole derivatives used clinically, i.e., the inhibition of sterol 14-α-demethylase, a microsomal cytochrome P-450 dependent enzyme system.[29] These compounds thus impair the biosynthesis of ergosterol for the cytoplasmic membrane and lead to the accumulation of 14-α-methyl sterols which may disrupt the close packing of acyl chains of phospholipids, impairing the function of membrane-bound enzymes and inhibiting growth.[29,30]

Table I: Inhibition of growth with derivatives **4-7** and clotrimazole **8** (at concentrations of 10 μM) of *Aspergillus* and *Candida spp.* after 48 hours, at 25°C.

| Compound | % Inhibition[a] | | |
|---|---|---|---|
| | *Aspergillus niger* | *Aspergillus flavum* | *Candida albicans* |
| **4** | 22.5±3.6 | 14.1±2.7 | 5.2±1.4 |
| **5** | 78.9±5.4 | 83.6±4.2 | 42.1±2.8 |
| **6** | 43.7±3.0 | 29.5±4.1 | 13.1±3.5 |
| **7** | 44.9±3.7 | 31.6±2.8 | 10.3±4.0 |
| **8** | 88.5±5.2 | 79.0±4.2 | 66.7±5.5 |

[a]Mean ± standard error from 10 plates.

Acknowledgments. This research was financed in part from the EU grant ERB CIPDCT 940051.

### References

1. Part 8 of this series: M.Brezeanu, M.Badea, D.Marinescu, N.Stanica, M.A.Ilies, and C.T.Supuran, *Rev.Roum.Chim.*, in press.
2. G.Kornis, "1,3,4-Thiadiazoles", in "Comprehensive Heterocyclic Chemistry", A.R.Katritzky Ed., Pergamon Press, 1984, Vol. 6, Part 4B, pp. 545-578.
3. M.Davis, *Org. Compd. Sulphur, Selenium, Tellurium* **1979**, *5*, 440-449.
4. K.Lu and T.Loo, *Cancer. Chemother. Pharmacol.*, **1980**, *4*, 275-282.
5. K.Kusterer and S.Szabo, *Eur. J. Pharmacol.*, **1987**, *141*, 7-15.
6. R.O.Roblin, and J.W.Clapp, *J.Am.Chem.Soc.*, **1950**, *72*, 4890-4892
7. T.H.Maren, *Physiol.Rev.*, **1967**, *47*, 595-782.
8. T.H.Maren, "The links among biochemistry, physiology and pharmacology in carbonic anhydrase mediated systems", in "Carbonic Anhydrase - From Biochemistry and Genetics to Physiology and Clinical Medicine", F.Botré et al. Eds., VCH, Weinheim, 1991, pp. 186-207.
9. a) C.T.Supuran, "Carbonic anhydrase inhibitors", in "Carbonic Anhydrase and Modulation of Physiologic and Pathologic Processes in the Organism", I.Puscas Ed., Helicon, Timisoara, 1994, pp. 29-111; b) T.H.Maren, B.W.Clare and C.T.Supuran, *Roum. Chem. Quart. Rev.*, **1994**, *2*, 259-282.
10. T.H.Maren, *J.Glaucoma*, **1995**, *4*, 49-62.
11. D.M.Woodbury, in "Antiepileptic Drugs: Mechanisms of Action", G.H.Glaser et al. Eds., Raven Press, New York, 1980, pp. 617-633.
12. I.Puscas and C.T. Supuran, "Farmacologia Clinica da Ulcer Peptica" in "Aparelho Digestivo", J. Coelho Ed., Rio de Janeiro, MEDSI, 1996, pp. 1704-1734
13. a) T.C.Downie, W.Harrison, E.S.Raper and M.A.Hepworth, *Acta Cryst.*, **1972**, *B28*, 1584-1590; b) E.S.Raper, R.E.Oughtred and I.W.Nowell, *Inorg.Chim.Acta*, **1983**, *77*, L-89-L-94; c) E.S.Raper, *Coord.Chem.Rev.*, **1994**, *129*, 91-156.
14. a) M.Brezeanu, R.Olar, G.Manole and C.T.Supuran, *Rev.Roum.Chim.*, **1992**, *37*, 425-431; b)

C.T.Supuran, F.Cimpoesu, G.Manole and M.Brezeanu, *Rev.Roum.Chim.*, **1992**, *37*, 959-964; c) C.T.Supuran, C.I.Lepadatu, R.Olar, A.Meghea and M.Brezeanu, *Rev.Roum.Chim.*, **1993**, *38*, 1509-1517.
15. a) U.Hartmann and H.Vahrenkamp, *Inorg.Chem.*, **1991**, *30*, 4676; b) S.Ferrer, J.G.Haasnoot, R.A.G. de Graaff, J.Reedijk and J.Borràs, *Inorg.Chim.Acta*, **1992**, *192*, 129-138; c) G.Alzuet, L.Casella, A. Perotti and J.Borràs, *J.Chem.Soc.Dalton Trans.*, **1994**, 2347-2351; d) G.Alzuet, J.Casanova, J.A.Ramirez, J.Borràs and O.Carugo, *J.Inorg.Biochem.*, **1995**, *57*, 219-234.
16. a) A.Antoniu, C.T.Supuran, and M.Brezeanu, *Rev.Roum.Chim.*, **1995**, *40*, 203-207; b) L. Sumalan, J.Casanova, G.Alzuet, J.Borràs, A.Castineiras and C.T.Supuran, *J.Inorg.Biochem.*, **1996**, *62*, 31; c) C.T.Supuran, *Metal Based Drugs*, **1995**, *2*, 327-330; d) C.T.Supuran, *Metal Based Drugs*, **1995**, *2*, 331; e) C.T.Supuran, *Metal Based Drugs*, **1996**, *3*, 25-30; f) J.Borras, T.Cristea and C.T.Supuran, *Main Group Met.Chem.*, **1996**, *19*, 339; g) J.Borras, J.Casanova, T.Cristea, A.Gheorghe, A.Scozzafava, C.T.Supuran and V.Tudor, *Metal Based Drugs*, **1996**, *3*, 143; h) K.N.Thimmaiah, G.T.Chandrappa, W.D.Lloyd and C.Parkanyi, *Inorg.Chim.Acta* **1985**, *107*, 1.
17. C.T.Supuran, M.Barboiu, C.Luca, E.Pop, M.E.Brewster and A.Dinculescu, *Eur.J.Med.Chem.*, **1996**, *31*, 596.
18. a) W.S.Sly, D.Hewett-Emmett, M.P.Whyte and R.E.Tashian, *Proc.Nat.Acad.Sci USA*, **1983**, *80*, 2752; b) W.S.Sly, S.Sato and X.L.Zhu, *Clin.Biochem.*, **1991**, *24*, 311.
19. C.T.Supuran and I.Puscas, "Carbonic Anhydrase Activators", in "Carbonic Anhydrase and Modulation of Physiologic and Pathologic Processes in the Organism", I.Puscas Ed., Helicon, Timisoara, 1994, pp. 112-145.
20. J.E.Bennett, "Antifungal agents", in "The Pharmacological Basis of Therapeutics", 8th Edition, A.G.Gilman, TW Rall, AS Nies and P Taylor Eds., Pergamon Press, New York 1990, pp. 1165-1181.
21. J.G.Horsfall, *Bot.Rev.*, **1945**, *11*, 357.
22. C.T.Supuran, G.Loloiu and G.Manole, *Rev.Roum.Chim.*, **1992**, *37*, 1181.
23. a) M.Forster and H.Vahrenkamp, *Chem.Ber.*, **1995**, *128*, 541; b) T.Matsukura, Y.Nishimura, T.Ohtani, M.Sawada and K.Shibata, *Chem.Pharm.Bull.*, **1990**, *38*, 3140; c) T.Gajda, B.Henry and J.J.Delpuech, *Inorg.Chem.*, **1995**, *34*, 2455; d) T.Gajda, B.Henry and J.J.Delpuech, *J.Chem. Soc.Faraday Trans.*, **1994**, *89*, 157.
24. K.Nakamoto, "Infrared Spectra of Inorganic and Coordination Compounds", Pergamon Press, New York, 1986, pp. 115-126.
25. A.B.P. Lever, "Inorganic Electronic Spectroscopy", Elsevier Publishing Co. , Amsterdam, 1984, pp. 178-196.
26. L.Sacconi, F.Mani and A.Bencini, "Nickel", in "Comprehensive Coordination Chemistry", G.Wilkinson, R.Gillard and J.McCleverty Eds., Pergamon Press, Oxford, 1987, Vol. 5, pp. 1-347.
27. L.Banci, A.Bencini, C.Benelli, D.Gatteschi and C.Zanchini, *Struct.Bonding*, **1982**, *52*, 37-79.
28. J.N.Galgiani, *Clin.Lab.Med.*, **1989**, *9*, 269.
29. H. Vanden Bossche, *Drug Dev Res*, **1986**, *8*, 287.
30. J.M.T.Hamilton-Miller, *Adv.Appl.Microbiol.*, **1974**, *17*, 109.

**Received: September 13, 1996 - Accepted: September 17, 1996 - Received in revised camera-ready format: September 24, 1996**

